# Machine Learning-Based Harvest Date Detection and Prediction Using SAR Data for the Vojvodina Region (Serbia)

**DOI:** 10.3390/s25072239

**Published:** 2025-04-02

**Authors:** Gordan Mimić, Amit Kumar Mishra, Miljana Marković, Branislav Živaljević, Dejan Pavlović, Oskar Marko

**Affiliations:** 1 BioSense Institute, University of Novi Sad, 21000 Novi Sad, Serbia; gordan.mimic@biosense.rs (G.M.); miljana.markovic@biosense.rs (M.M.); branislav.zivaljevic@biosense.rs (B.Ž.); dejan.pavlovic@biosense.rs (D.P.); oskar.marko@biosense.rs (O.M.); 2 National Spectrum Centre, Aberystwyth University, Penglais, Aberystwyth SY23 2JH, UK; 3 Centre of Space Technology, AGH University of Science and Technology, 30-059 Krakow, Poland

**Keywords:** SAR, Sentinel-1, Google Earth Engine, machine learning, harvest dates, agricultural production

## Abstract

Information on the harvest date of crops can help with logistics management in the agricultural industry, planning machinery operations and also with yield prediction modelling. In this study, the determination and prediction of harvest dates for different crops were performed by applying machine learning techniques on C-band synthetic aperture radar (SAR) data. Ground truth data were provided for the Vojvodina region (Serbia), an area with intensive agricultural production, considering winter wheat, maize and soybean fields with exact harvest dates, for the period 2017–2020, including 592 samples in total. Data from the Sentinel-1 satellite were used in the study. Time series of backscattering coefficients for vertical–horizontal (VH) and vertical–vertical (VV) polarisations, both from ascending and descending orbits, were collected from Google Earth Engine. Clustering of harvested and unharvested fields was performed with Principal Component Analysis, multidimensional scaling and t-distributed Stochastic Neighbour Embedding, for initial cluster visualization. It is shown that the separability of unharvested and harvested data in two-dimensional space does not depend on the selected method but more on the crop itself. Support Vector Machine and Multi-layer Perceptron were used as classification algorithms for harvest detection, with the former achieving higher accuracies of 79.65% for wheat, 83.41% for maize and 95.97% for soybean. Finally, regression models were developed for the prediction of the harvest date using Random Forest and the long short-term memory network, with the latter achieving better results: an *R*^2^ score of 0.72, mean absolute error of 6.80 days and root mean squared error of 9.25 days, for all crops considered together.

## 1. Introduction

Nowadays, Earth Observation (EO) is applied for the monitoring of agricultural fields, to provide insight into crop development [1], plant health status [2], canopy water content [3] and biomass productivity [4]. Crop phenology represents the timing of different stages of the plant growth and development, such as leaf emergence, flowering or maturation, and their relationship with the environmental conditions through the growing season [5]. Satellite remote sensing is particularly used for monitoring of crop phenology [6] since detection of specific phenological stages is of interest in agricultural management. Different approaches are used on time series of canopy spectral reflectance, vegetation indices or backscatter intensity in order to detect key dates during the growing season, such as: threshold-based methods [7], derivative methods [8] and shape model methods [9]. Deployment of the specific approach highly depends on the amount of ground truth data from the fields.

Information on the harvest date of crops can help with logistics management in the agricultural industry, planning machinery operations and also with yield prediction modelling [10]. Remote sensing of the large-scale harvest detection of various crops, by using satellite imagery, can be found in the current scientific literature, although it has not been fully explored so far. The time series of backscattering for Vertical–Horizontal (VH) polarisation from Sentinel-1 and the Enhanced Vegetation Index (EVI) from Sentinel-2 were proposed to recognise rice planting and heading dates, respectively, while the Normalised Difference Yellow Index (NDYI) was utilised to detect the rice harvest date [11]. Tracking of asparagus development and the estimation of the harvest date were performed with VH backscatter intensity from Sentinel-1 and the Green Normalised Difference Vegetation Index (GNDVI) derived from Sentinel-2 [12]. Given that wheat is a worldwide staple food, monitoring of winter wheat phenology has been explored in several studies. Based on Landsat 8 images, the changes in the EVI, Normalised Difference Vegetation Index (NDVI) and Normalised Difference Water Index (NDWI) were analysed at different growth stages of winter wheat [13], including the harvest date [14]. In [15], the authors even analysed the optimal set of bands of Sentinel-2 among 13 available bands to predict the most accurate time for the harvesting of wheat crops, where Red, Blue and NIR bands are found to be optimal. The phenology of soybean, an industrial crop vastly cultivated for oil and protein, has also been a research focus. Different tools were analysed for the estimation of sowing and harvest dates using the NDVI and EVI2 (two-band EVI) from Sentinel-2 [16], and the EVI from MODIS [17,18]. One of the first attempts to assess the optimal harvesting date in maize fields indirectly used surface reflectance data acquired from HJ-1 satellites [19].

The main issue of utilising optical sensors for harvest date detection is that there may be gaps in the observations acquired on cloudy days; hence, as a result, there are a reduced and irregular number of days between observations in a time series. Since the synthetic aperture radar (SAR) provides all-weather and all-day imagery, potentially it is more suitable for this task. A comprehensive review of radar remote sensing of agricultural canopies, including crop phenology monitoring, is given in [20]. Recently, Sentinel-1 SAR images were mostly exploited for this purpose. Time series of the coherence of vertical transmit and vertical receive (VV) polarisation, and the backscattering coefficient ($\sigma^{0}$) in vertical transmit and horizontal receive (VH) polarisation, were used to create an algorithm which finds a step-like increase in coherence that occurs after the harvesting, and an additional check of potential wheat harvest dates was carried out by using the threshold values of $\sigma_{VH}^{0}$ depending on vegetation height [21]. Similar methodology was applied for soybean fields, comparing a maximum difference of ascending $\sigma_{VH}^{0}$ between consecutive dates of observation with the threshold value of the Radar Vegetation Index (RVI), where the former method showed better performance for harvest detection [22]. Another algorithm was accomplished by analysing both the interferometric coherence (IC) and $\sigma_{VH}^{0}$ time series in an individual way for soybean and maize. Based on the latter, separate criteria were adopted [23]. Additionally, harvest dates for different crop fields were determined by change detection in time series of both IC and the backscattering coefficient in VV and VH polarisation [24].

The above-mentioned studies were investigating a step-like increase in the signal, determination of the threshold value or change detection in time series, while none of them exploited the use of machine learning methods on SAR data for predictive analytics of the harvest date. The objective of this study was the determination of the harvest dates for winter wheat, maize and soybean fields in Vojvodina (Serbia), a region with intensive agricultural production. For this purpose, time series of the SAR backscattering coefficient at VH and VV polarisation, from both ascending and descending Sentinel-1 orbits, were exploited. For the first time, data analysis was performed by applying various machine learning (ML) techniques. This work has three main contributions. First of all, we applied different ML algorithms of harvest date estimation by conducting cluster analysis, followed by classification methods, and finally by regression models. Secondly, we showed how clustering can be used to gain rough estimation on the separability of unharvested and harvested data for certain crops, and thus to obtain a reasonable expectation of the performance of classification methods. Lastly, the study is rich given the amount of ground truth data we have used thanks to our industrial partner. This makes the study much more reliable.

The rest of the paper is organised as follows. Section 2 describes the data we have used and our data preprocessing steps. Section 3 presents the clustering, classification and regression algorithms used in this study. Section 4 discusses the results, and the paper ends with a concluding section.

## 2. Materials and Methods

### 2.1. Study Area

**Figure 1:**
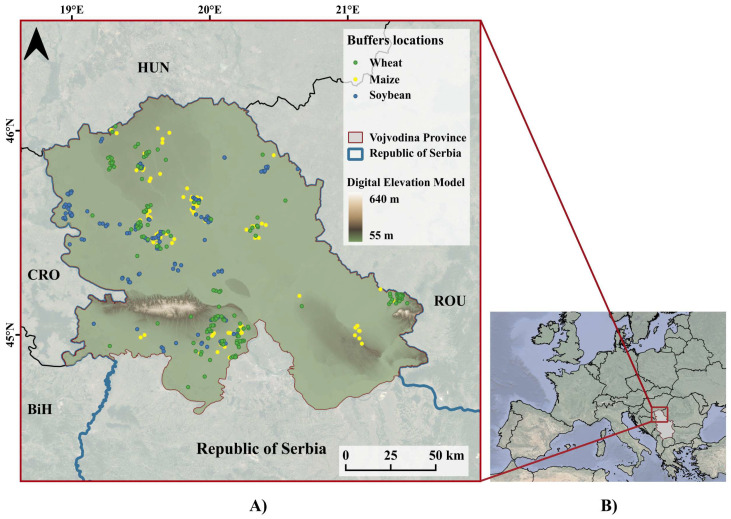
Geolocation of ground truth data in the study area (**A**) and the position of the study area in Southeastern Europe (**B**).

The Vojvodina region is the northern part of the Republic of Serbia, with a total area of 2,150,000 ha, located on the southern edge of the Pannonian Plain [25] (Figure 1). The terrain is mostly flat and the Digital Elevation Model (DEM) of the study area, with a resolution of 10 m, is given in Figure 1A. The area is characterised by a temperate climate, fully humid with a hot summer [26]. The average annual temperature is 11.1 °C, while the average amount of precipitation is 606 mm [27], with maximum precipitation in June. The Vojvodina region is characterised by predominantly agricultural land (83%), most of which is cropland (77%), with intensive agricultural production [28], where wheat, maize and soybean are the most cultivated crops, according to the Statistical Office of the Republic of Serbia [29]. The agricultural fields in the study area are mainly rain-fed with just a small percentage of them being irrigated [30].

### 2.2. Data

#### 2.2.1. Data Acquisition

Ground truth data needed for this research were received from the agricultural company and were obtained as geolocated points (latitude and longitude) representing a production parcel. For each parcel, information on sowing and harvesting dates was additionally provided, from 2017 to 2020 for winter wheat, and from 2018 to 2020 for maize and soybean. The data for different years were combined together. Figure 1A shows the location of the points for each crop in the study area. To ensure relevant representation of each parcel, buffers of 50 m radius were created around each point and used in further analysis. However, after visual inspection of Sentinel-2 RGB images, some of the buffers were overlapping boundaries to neighbouring crops due to a small parcel size or the location of a point near the border. Those buffers were excluded from the analysis, resulting in the number of final buffers for winter wheat being 203, for maize 214 and for soybean 175 (representing 592 samples in total). Considering different years, the number of samples was as follows: 39 (wheat only) for 2017, 193 for 2018, 181 for 2019 and 179 for 2020, with various weather conditions in the study region [31].

Sentinel-1 C-band SAR images were downloaded from the Google Earth Engine (GEE) platform, which contains Sentinel-1 Ground Range Detected (GRD) scenes, calibrated and ortho-corrected images. The relative orbits covering the study region for ascending passes are 73, 175 and 102, while for descending passes they are 51, 153 and 80. The size of the pixel was 30 m due to the resampling procedure. Thus, the number of pixels inside a buffer varied between 14 and 21, due to its circular shape and the alignment with the Sentinel-1 pixels. For each buffer, backscattering coefficients ($\sigma^{0}$) at VH and VV polarisation were extracted from both ascending and descending orbits. This created time series of $\sigma_{VV}^{0}$ and $\sigma_{VH}^{0}$ during the growing season, with the data every six days at a minimum, while for some parcels, data were available even more frequently due to overlapping scenes. Note that backscattering coefficients were not corrected for the effects of the incidence angle since the signal was not influenced by the flat terrain (Figure 1A) and localized buffers. The dates of the images used in the study are given in the Supplementary Materials.

#### 2.2.2. SAR Data Preprocessing

**Figure 2:**
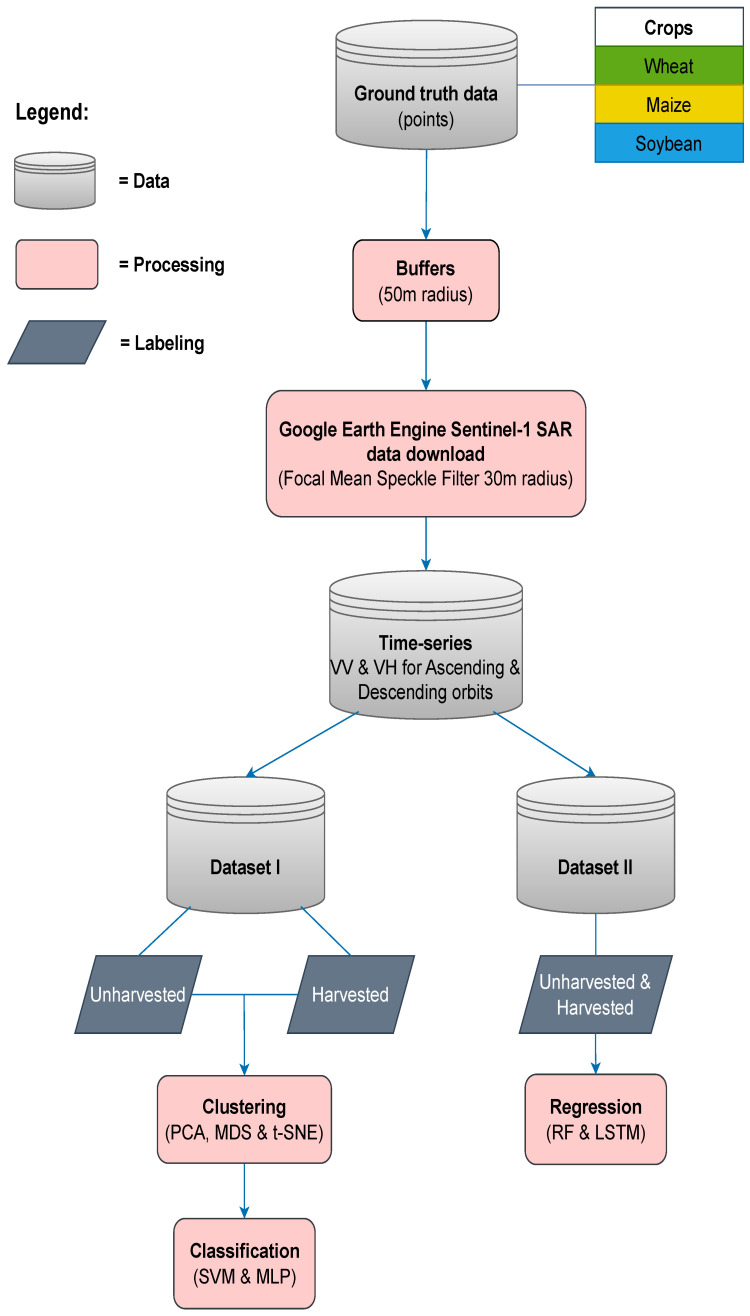
The flowchart presenting data processing.

**Figure 3:**
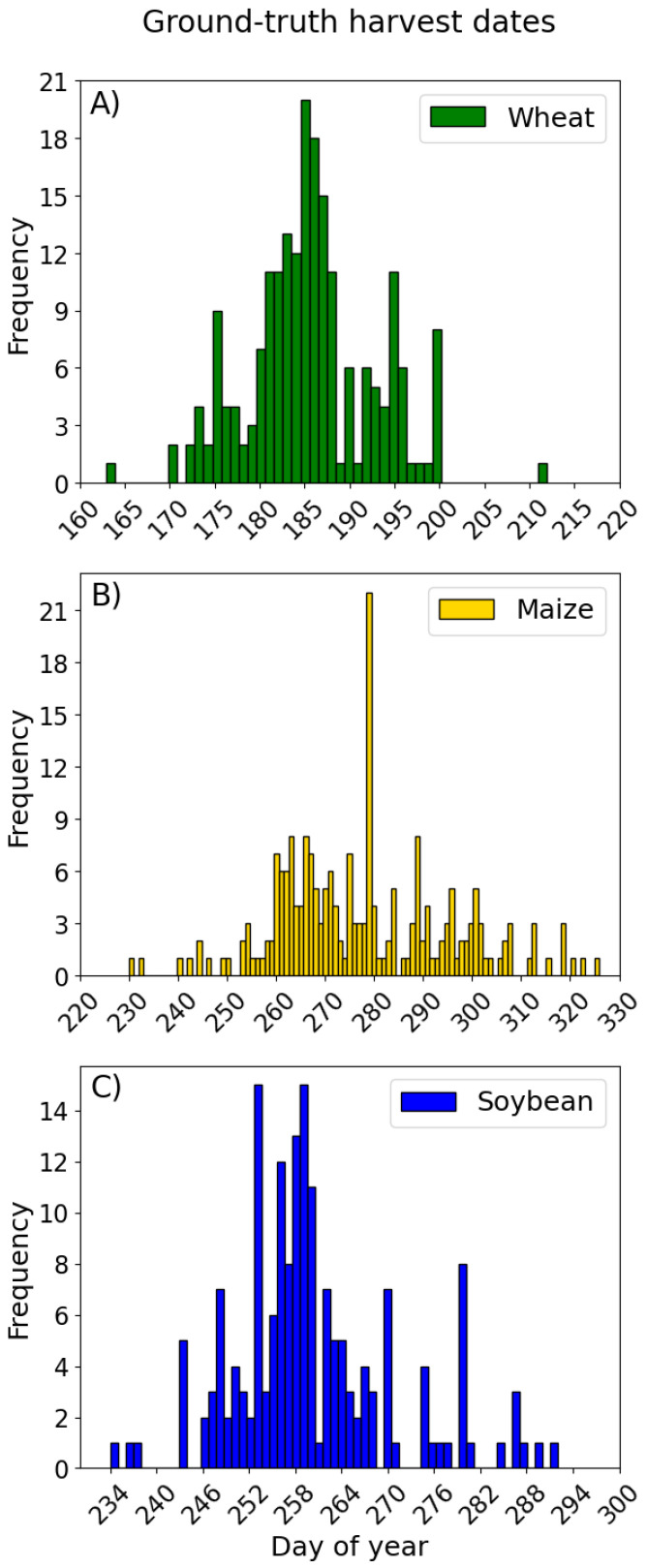
Temporal distribution of harvest dates in the ground truth data for wheat (**A**), maize (**B**) and soybean (**C**).

Prior to downloading the data from GEE, the Focal Mean Speckle Filter was applied to limit the noise within a 30 m radius circle [32]. In terms of experiments, we plan to work on clustering, followed by classification and finally harvest date prediction. Accordingly, two datasets were created (Figure 2). The first one was prepared for cluster analysis and classification experiments, where data were separated into two groups and labelled as unharvested or harvested. Each sample consisted of four features referring to VH and VV data observed at ascending and the closest date of descending acquisition. The groups were comprised of an equal number of samples across all crops. Based on the ground truth data (Figure 3), the harvest period for each crop was identified in the following time windows: May 1st to July 31st for winter wheat, August 15th to November 30th for maize and August 15th to October 31st for soybean. The second dataset was prepared for the regression models to predict the harvest date, including time series with a six-day time step, since inputs with the same length are required for the regression algorithms used in this study. Hence, the data from 15 dates were used for every parcel. For each crop, a three-and-a-half-month period before the earliest harvest date was defined as input data: March 1st to June 15th for winter wheat and May 1st to August 15th for both maize and soybean. The target value for prediction was defined as the number of days from the last date of observations within a defined time window to the harvest date at the given parcel.

### 2.3. Machine Learning Algorithms

In this study, we conducted three stages of investigation, viz. as follows

- Clustering analysis, which was performed in the initial phase to better understand the data. The aim was to have an understanding of the data structure and, based on that, an intuitive understanding of the levels of performances we could expect from the harvest date detection exercise.
- Classification analysis, where the aim was to classify the data from pre- and post-harvest periods. This is motivated by the assumption that the data should have harvest-related information in a hyper-space. An efficient binary classification of pre- and post-harvest data would show the presence of harvest-related information in the data.
- Regression analysis, where we use ML algorithms to predict the harvest date based on remote sensing data from the past. This is the most challenging task and the actual challenge we are working on in this paper. Having the previous two stages makes our work thorough and methodical.

The study was implemented using Python, v3.9 (Anaconda Inc., Austin, TX, USA) and open-source libraries (such as TensorFlow, Scikit-learn, etc.). In this section, we shall first explain the algorithms we have used for all these three stages. Following this, we shall mention the performance metrics used for the models’ evaluation. Finally, we will list the hyper-parameters of these algorithms which we have found to be the most suitable for our tasks.

#### 2.3.1. ML Algorithms Used

In the cluster analysis phase of our work, we used three algorithms, viz., PCA, MDS and t-SNE. The motivation behind using these particular algorithms was as follows. We wanted to test clustering algorithms which could extract clusters for a linearly clusterable dataset followed by cases where it is increasingly difficult to visualise the clusters. Intuitively speaking, for cases where a linear clustering algorithm (like PCA) is able to distinguish the clusters, we should be able to use relatively naive classification and regression algorithms as well.

Principal Component Analysis (PCA) is a linear dimensionality reduction technique using singular value decomposition of the data to project the data to a lower dimensional space where the dimensions are uncorrelated as well. It is used to decompose a multivariate dataset in a set of successive orthogonal components that explain a maximum amount of the variance [33].

Multidimensional scaling (MDS) seeks a low-dimensional representation of the data in which the distance between data points is preserved as much as possible, corresponding with the original high-dimensional space. In general, MDS is a technique used for analysing similarity or dissimilarity in data, trying to model them as distances in a geometric space [34].

For cases needing extremely nonlinear mapping of data space to make the clustering apparent, t-distributed Stochastic Neighbour Embedding (t-SNE) is used, since it converts affinities of data points to probabilities. The affinities in the original space are represented by Gaussian joint probabilities and the affinities in the embedded space are represented by Student’s t-distributions [35]. This allows t-SNE to be particularly sensitive to local structure, which might be beneficial to visually disentangle a dataset that comprises several manifolds at once.

In our work, we tried two classification algorithms. The motivation, like our experimentation with clustering, is to try a classical method and one based on an artificial neural network. We chose SVM and MLP for this phase.

Support Vector Machine (SVM) is a supervised kernel-based learning method, relying on a maximal margin hyperplane, that can be used for classification in high-dimensional spaces. SVMs are still effective in cases where the number of dimensions is greater than the number of samples and also memory efficient since they use a subset of training points in the decision function [36].

Multi-layer Perceptron (MLP) is a supervised learning algorithm capable of learning nonlinear relations between features while transforming the input to the predicted output. The learning process assumes optimisation of the parameters (weights and biases) in order to minimise the error between the model output and ground truth data [37]. The model training is performed iteratively since at each time step, the partial derivatives of the loss function with respect to the model parameters are computed with the aim to update those parameters in order to minimise the value of a given loss function [38]. The MLP algorithm requires tuning a number of hyper-parameters such as the number of hidden neurons, layers and iterations, while different random weight initialisation can lead to different validation accuracy.

Prediction of the harvest date is a regression task. For this, like for the classification phase, we chose to experiment with one classic algorithm and one algorithm based on a neural network. We decided to go with Random Forest and Recurrent Neural Network algorithms. The choice of RF is based on the fact that a number of use cases with limited training data have reported RF to be an efficient algorithm.

Random Forest (RF) is a meta estimator that fits a number of decision tree regressors on various sub-samples of the dataset, where trees in the forest use the best split strategy. RF uses averaging to improve the prediction accuracy and control overfitting [39].

Recurrent Neural Network (RNN) represents an artificial neural network architecture characterised by connections between units that create a directed cycle, facilitating the demonstration of dynamic temporal behaviour [40]. The key distinction of RNN from feedforward neural networks, such as MLP, lies in the inclusion of feedback loops, which generate recurrent connections within the unfolded network. Nonetheless, due to the issue of vanishing gradients, training deep RNN models, using commonly employed activation functions, poses a significant challenge [41]. For that reason, long short-term memory (LSTM) architecture has been introduced, which substitutes the nonlinear units found in traditional RNNs with LSTM cells, comprising the internal mechanisms, called gates. Input, output and forget gates represent fundamental components of RNN-LSTM models that serve to regulate the flow of information and select which information is relevant to remember, thus being able to capture long-distance dependencies [42]. In recent years, there has been a wide use of RNNs in various machine learning fields that involve sequential data [40], which also represented a main premise behind employing LSTM models for harvest date prediction.

#### 2.3.2. Performance Metrics

No free-lunch theorem dictates that no algorithm is universally better than another. Hence, it is crucial to define the figures of merit based on which we want to compare our algorithms. In our study, we work on three stages of experimentation, viz., clustering, classification and regression. Based on this, we define three sets of performance metrics.

For the analysis of the clustering methods, visual inspection of the graphical results was performed to better understand the data.

The results of the classification models were evaluated using standard metrics such as overall accuracy (Acc), precision (P), recall (R) and F1 score [43]. Considering conventional notation on true positive ($T_{p}$), true negative ($T_{n}$), false positive ($F_{p}$) and false negative ($F_{n}$) samples, the metrics are defined as follows.

(1) $$Acc=\frac{T_{p}+T_{n}}{T_{p}+T_{n}+F_{p}+F_{n}}$$

(2) $$P=\frac{T_{p}}{T_{p}+F_{p}}$$

(3) $$R=\frac{T_{p}}{T_{p}+F_{n}}$$

(4) $$F1=2\times \frac{P\times R}{P+R}$$

Assessment metrics of the regression models were mean absolute error (MAE) and root mean squared error (RMSE), both given as the number of days, and coefficient of determination ($R^{2}$). Considering that *n* is the number of samples, *M* stands for modelled, while *O* stands for observed values and $\hat{O}$ is the observed average, the metrics are given below.

(5) $$MAE=\frac{1}{n}\sum_{i=1}^{n}\left|O_{i}-M_{i}\right|$$

(6) $$RMSE=\sqrt{\frac{1}{n}\sum_{i=1}^{n}{\left(O_{i}-M_{i}\right)}^{2}}$$

(7) $$R^{2}=1-\frac{\sum_{i=1}^{n}{\left(O_{i}-M_{i}\right)}^{2}}{\sum_{i=1}^{n}{\left(O_{i}-\hat{O_{i}}\right)}^{2}}$$

#### 2.3.3. Hyper-Parameters Used

For the majority of parameters in each clustering method, default parameter settings were utilised. However, for some of them a specific configuration was customised for a detailed analysis. The number of components to keep was set to 2 in all three clustering cases. Other than that, PCA was conducted with default settings, while MDS and t-SNE were performed with three and two custom parameters, respectively (Table 1).

In this study, we utilised the GridSearchCV module from Scikit-learn to optimise SVM and MLP classifiers through a grid search with a 5-fold cross-validation. For the SVM classifier, we explored hyper-parameters including ‘C’, which expresses regularisation strength [0.1, 1, 10, 100]; ‘gamma’, affecting the impact of individual training samples [‘scale’, ‘auto’, 1, 0.1, 0.01, 0.001]; and ‘kernel’, defining the type of kernel function [‘rbf’, ‘linear’, ‘poly’]. Similarly, for the MLP classifier, we investigated ‘hidden layer sizes’ to determine the architecture of hidden layers, ranging from single-layer configurations like (64) and (128) to deeper architectures such as (256, 256). Additionally, activation functions were varied [‘identity’, ‘logistic’, ‘tanh’, ‘relu’]. The MLP classifier was configured with a maximum iteration limit of 1000, an adaptive learning rate scheme and a fixed random state for reproducibility. Through a grid search, we identified the optimal combination of hyper-parameters that achieved the highest cross-validated accuracy score (Table 2), which was then used to instantiate optimised SVM and MLP classifiers for subsequent analyses. Utilising a subset of features and standard scaling for data normalisation, the models’ performances were evaluated through a 5-fold cross-validation. The models’ accuracy, precision, recall and F1 score were calculated from a summarised confusion matrix aggregated across all folds, using a binary average to measure these metrics. It is important to state here that for both algorithms, four data-driven classification models were created in total. The first model was built by using the data for all crops, and then, three additional models were built using the data for the specific crop only.

The RF regression model was optimised through a grid search with a 5-fold cross-validation using a predefined hyper-parameters grid. The procedure was iterated five times, resulting in the creation of 25 unique models for each parameter combination. Explored hyper-parameters include the number of estimators [50, 100, 200, 500], the maximum depth of each tree [none, 10, 15, 20], the minimum number of samples required to split an internal node [2, 5, 10] and the minimum number of samples required to be at a leaf node [1, 2, 4]. Stratified K-fold cross-validation with five splits is employed to ensure a balanced representation of crops across folds. Within each fold, the dataset undergoes preprocessing, including feature scaling. RF regressor is instantiated, and a grid search with cross-validation is performed to find the optimal combination of hyper-parameters. The parameter combination exhibiting the highest $R^{2}$ was chosen for subsequent analyses. The final model configuration consists of 200 estimators, a maximum depth of 10 nodes, a minimum of 5 samples per split and a minimum of 4 samples per leaf (Table 3).

In order to optimise the LSTM architecture, different numbers of LSTM layers [1, 2, 3], numbers of hidden units [64, 128, 256], probabilities of the dropout layers [0, 0.25, 0.5] and activation functions [tanh, relu] were thoroughly explored. A hyper-parameter search was performed by splitting the dataset in a stratified manner using a 5-fold cross-validation procedure. Considering the stochastic nature of the optimisation process during model training, the whole procedure was repeated five times, thus generating 25 distinct deep learning models per parameter combination. The parameter combination with the highest average $R^{2}$ was selected for further analyses. The early stopping criteria based on the validation loss was used in order to fight the model overfitting [44], with the optimum model being selected from the training cycle after the completion of the process. The network was trained to a maximum of 1000 epochs with early ending when validation loss reached a plateau at a patience of 200, whereas batch size was set to 32. RMSprop [45] was utilised as an optimisation algorithm at a learning rate of 1 × 10^−2^. The proposed architecture was defined by two segments, namely, a feature extractor segment configured utilising LSTM layers followed with BatchNormalisation and DropOut layers and a classifier segment consisting of a single fully connected layer. An architecture with two LSTM layers, comprising of 128 hidden units and a tanh activation function and dropout probability of 0, yielded the best average performance in terms of $R^{2}$ (Table 3), with a more detailed description on model performance being reported in Section 3.

It can be noted here that for both the algorithms, the data-driven model was trained using data for all the crops. However, the model was validated not just for all crops together but also for each crop separately.

## 3. Results

### 3.1. Clustering

**Figure 4:**
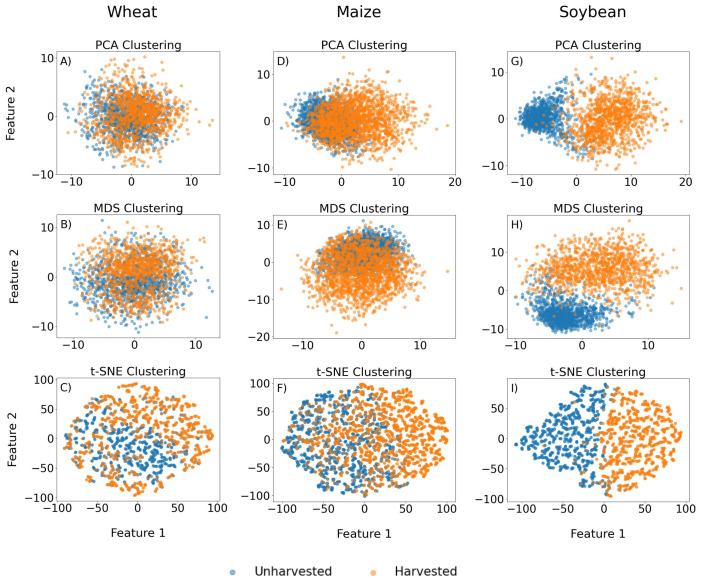
The results of clustering for: wheat with PCA (**A**), MDS (**B**) and t-SNE (**C**); maize with PCA (**D**), MDS (**E**) and t-SNE (**F**); soybean with PCA (**G**), MDS (**H**) and t-SNE (**I**).

The results of three different clustering methods, i.e., PCA, MDS and t-SNE, are given in Figure 4. It is shown that the separability of unharvested and harvested data in two-dimensional space does not depend on the selected method but more on the crop itself. For wheat (Figure 4A–C), data points are mainly overlapping; thus, it is not possible to distinguish between the clusters. In the case of maize (Figure 4D–F), although the points are overlapping, there are some zones where only harvested data are present, i.e., localised separability exists. On the other hand, the clusters are clearly distinguished for soybean (Figure 4G–I), with only several points overlapping. In this example, the t-SNE algorithm proved to have more discriminatory power compared to MDS and PCA, indicating non-linearity in soybean data.

### 3.2. Classification

Several classification models were developed for the three different crops. The first model was built by using the data for all crops, called the general model. The performance metrics for both the SVM and MLP algorithms show that they are quite effective in classifying harvested fields, with both models achieving an overall accuracy (Acc) greater than 80% across all crops, and having quite similar values of precision, recall and F1 score (Table 4). Additionally, independent models were built using the data for specific crops only, and they show variations in accurately classifying harvested fields. The SVM model slightly outperforms the MLP model in overall accuracy for wheat classification, achieving 79.65% compared to 76.69%. However, SVM shows lower precision, indicating a higher rate of false positives. This suggests that while the SVM model is better at identifying wheat harvest, it also tends to misclassify some unharvested fields as harvested. Conversely, its higher recall implies a greater ability to correctly identify true instances of harvested wheat fields among all wheat samples. In maize classification, while the MLP model shows a marginal accuracy advantage, the SVM model demonstrates superior precision of 90.24%, and the MLP model has slightly better recall. The close F1 scores for maize reflect their comparable effectiveness, suggesting both models are suitable for maize-related applications, depending on whether precision or recall is prioritised. Soybean classification highlights the models’ exceptional performance, with both algorithms achieving over 95% on all metrics and SVM holding a slight edge.

Additionally, we tested the general model on each specific crop and the performance metrics are given in Table 5. By comparing the results with those obtained with the specific models (Table 4), it can be seen that the general model is slightly under-performing on the specific models, both for SVM and MLP, on average around 5% for all the metrics. Thus, it can be used for the specific crops but better results are achieved with the specific models.

**Figure 5:**
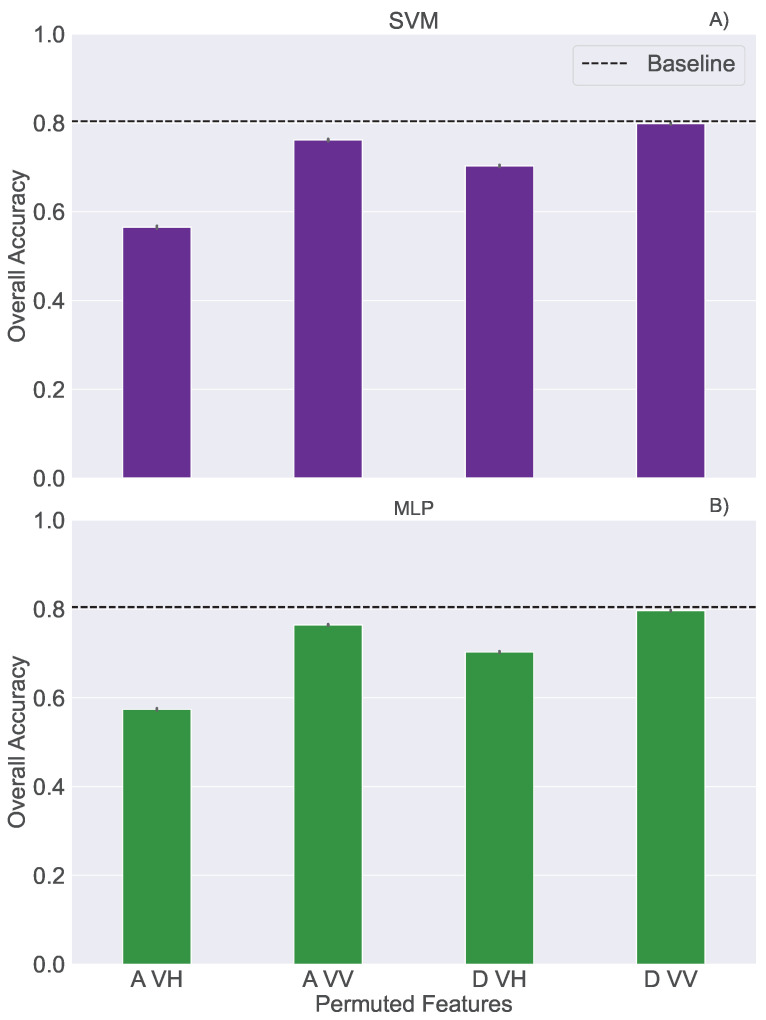
Performance of classification models: (**A**) SVM and (**B**) MLP, after the random permutation test.

We carried out the random permutation test to explore what polarisation channels and orbits are more relevant for harvest date detection. These tests were performed for the general models applied on all crops together, considered here as a baseline. Permutations were performed five times for each individual feature and corresponding results are presented in Figure 5. A greater decrease in model performance suggests that the feature provides more valuable information. It is shown that VH polarisation data from an ascending orbit are the most important feature for the classification task, which is in accordance with previous studies [22]. VV polarisation features from both orbits expressed less importance. The consistency between the results obtained with SVM and MLP (Table A1 in Appendix A) indicates that they are algorithm agnostic, highlighting the importance of the information provided in the dataset

### 3.3. Regression

As mentioned, the optimal RF model architecture was determined based on the highest average performance across a series of metrics, utilising 25 distinct models generated through a 5-fold cross-validation over five iterations. The overall average performance across all crops was observed to be 0.47, 13.25 and 9.52 for the $R^{2}$, RMSE and MAE metrics, respectively, suggesting that the model achieves a moderate level of predictive accuracy. The average $R^{2}$ values for wheat, maize and soybean were −0.04, 0.11 and −0.45, respectively. Similarly, the average RMSE and MAE values for given crops are 9.97 and 6.17 (wheat), 16.19 and 12.92 (maize), and 11.37 and 9.02 (soybean). The RMSE and MAE metrics further illustrate the challenges faced by the model, with relatively high error rates indicating that the model’s predictions are often far from the actual values. The variability observed in $R^{2}$ values, represented with the average standard deviation of $R^{2}$, being considerably more pronounced for soybean (0.77) than those obtained for wheat (0.24) and maize (0.25).

As discussed above, the optimal LSTM architecture was selected based on the highest average performance in terms of the $R^{2}$ metric obtained utilising 25 distinct deep learning models (five folds and five iterations). The average performances among all folds and iterations for all crops are 0.66, 10.36 and 7.51 regarding $R^{2}$, RMSE and MAE, respectively. In terms of individual performance per crop, the proposed model architecture most confidently predicted the harvest date of maize, while soybean represented the most challenging crop for a harvest date prediction. An average $R^{2}$ per crop is 0.22, 0.40 and 0.14 for wheat, maize and soybean, respectively. On the other hand, the average RMSE and MAE for the given crops are 6.47 and 4.88 (wheat), 13.33 and 10.04 (maize), and 9.52 and 7.46 (soybean). The average standard deviation of $R^{2}$ for wheat and maize is 0.11 and 0.12, respectively, whereas for soybean this equals 0.29. The obtained results indicate that the LSTM algorithm outperformed RF in the task of harvest date prediction. The best performing LSTM model, based on the highest $R^{2}$ score among the models comprising the optimal architecture, represents the proposed solution by authors for this demanding task. Its overall performance, as well as individual performances per crop, are shown in Table 6.

**Figure 6:**
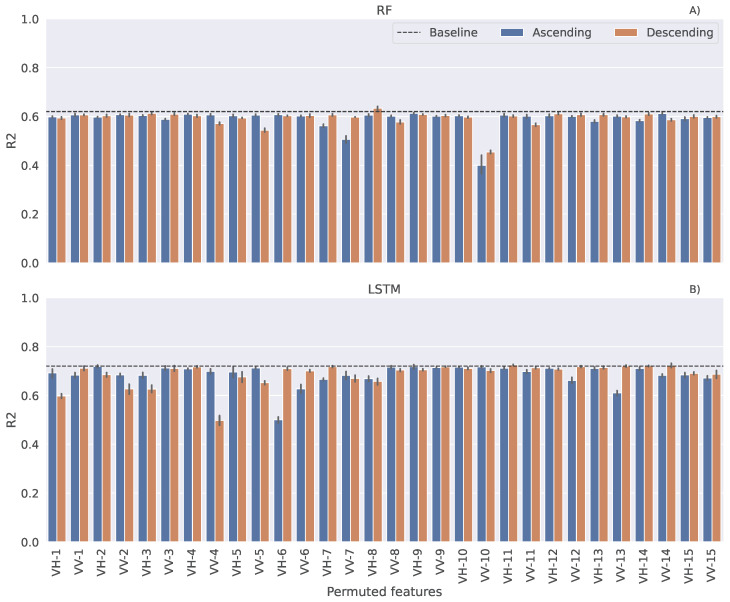
Performance of regression models: (**A**) RF and (**B**) LSTM, after the permutation feature importance test.

We explored what polarisation channels and dates are more relevant for harvest date prediction by using the permutation feature importance test. This method involves randomly shuffling the values of one feature at a time and observing how this disruption impacts the model’s performance. It was applied on the best regression models, considered here as a baseline. Permutations were performed five times and the corresponding average performance for each individual feature is presented in Figure 6. Dates are chronologically ordered, meaning that number 1 refers to the earliest date and 15 the latest. Note that a greater decrease in model performance suggests that the feature provides more valuable information for the particular task and has a greater influence on the model’s predictions. For the RF model, VV from both orbits at the 10th date were strictly recognized as the most important features. However, in the case of the LSTM model, several features in the first half of the season from both polarisations and orbits highly influenced the performance, hence containing the relevant information for the learning process. Since each LSTM layer has memory cells, it considers a temporal sequence within the data, which is not the case with the RF algorithm.

## 4. Discussion

**Figure 7:**
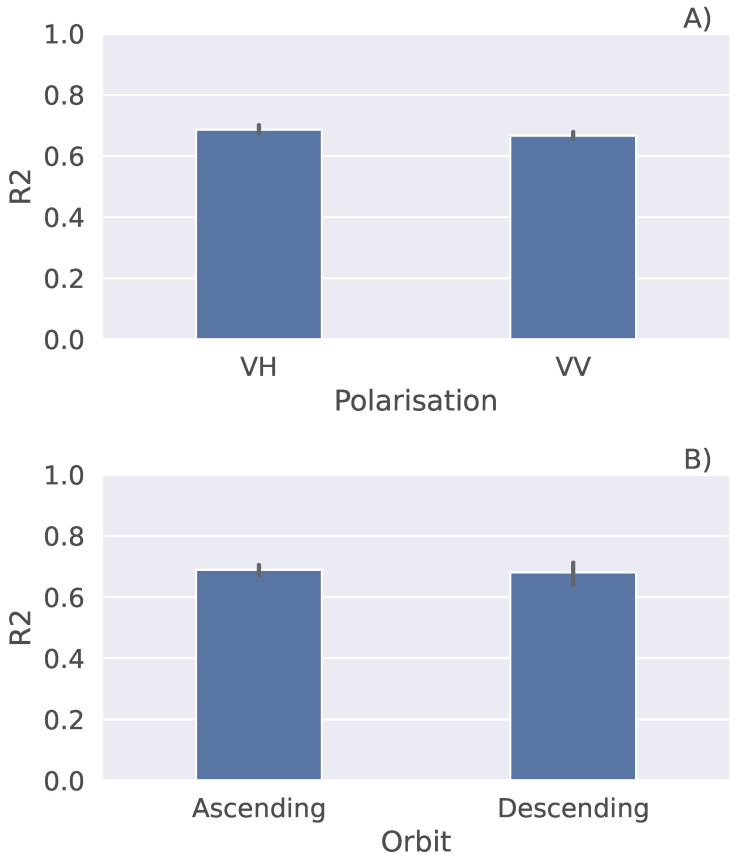
The LSTM model performance with only a polarisation (**A**) or an orbit (**B**).

In order to explore the performance of the LSTM model in more detail, we carried out several experiments. First, we reduced the number of features used to run the model by selecting only a polarisation or an orbit. In one experiment, we used VH polarisation, while in the other, we used VV polarisation (Figure 7A). This decreased the $R^{2}$ score of the model to approximately 0.6 in both cases. Additionally, we ran the model with the data from the ascending orbit, and later from the descending orbit (Figure 7B). It can be noticed that $R^{2}$ decreased, being slightly above 0.6 for both the ascending and descending orbits, where the latter has a slightly higher standard deviation represented with the error bar.

**Figure 8:**
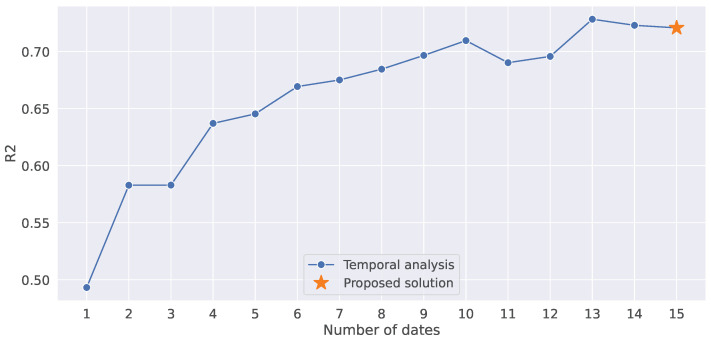
The LSTM model performance when using the data of different lengths.

Second, we used the time series of different lengths as input to run the model, by sequentially reducing the number of dates one by one (Figure 8). Overall, it can be seen that there is a decreasing trend of the $R^{2}$ score when shortening the length. There is just a slight decrease when only 10 dates are used, there are no big differences between 9 and 6 dates, while there is a steep decrease when fewer than 5 dates are used. This is highly in accordance with the results of the permutation feature importance test (Figure 6B), showing that the model is learning relevant information from the features in the first half of the season.

Regrading the prediction of the harvest date for winter wheat, the LSTM model with an MAE of 4.87 and RMSE of 5.86 outperformed the MAE of 6.5 days and RMSE of 8 days for spring cereals (mainly wheat) in Kazakhstan, also obtained by using SAR data [21]. With overall accuracy above 80%, both classification models developed in this study for all crops outperformed the result of 56% for harvest date identification for cereals, soybean and maize, among other crops in Canada, using InSAR data [24]. This performance metric achieved for all three crops underscores the models’ robustness and potential applicability for diverse crops. The overall accuracies of the classification models developed for each crop separately, being 79.65% for wheat, 83.41% for maize and 95.97% for soybean, are in line with the results of the clustering analysis and observed separability of data points in 2D space. The outstanding results particularly for soybean could imply distinct or more separable features compared to the other crops, offering insights for focused soybean research or tailored agricultural practices. This is in accordance with the results for the harvest detection of soybean and maize in Argentina achieved with SAR data, where overall accuracy was up to 93% for both crops [23]. This is, mostly, because of the difference in the physical features of the crops before and after harvest. In one of our future works, we intend to expand in this direction and develop phenomenological ML models that leverage the physical differences in the crops before and after harvest in order to enhance the performance of the ML models.

In the near future work, it would be interesting to test the SVM and LSTM models for harvest date detection and prediction by conducting transfer learning in some other agricultural regions in Europe. The suitable option for that might be the Netherlands, which has quite similar terrain to that in Vojvodina, and where wheat and maize are grown as well. The future work might comprise data fusion of Sentinel-1 SAR and Sentinel-2 optical data to investigate the potential benefits of including more data in visible, NIR and SWIR parts of the electromagnetic spectrum in the analysis.

## 5. Conclusions

In this study, determination and prediction of harvest dates for winter wheat, maize and soybean were performed by applying various machine learning techniques on Sentinel-1 SAR data. The cluster analysis with PCA, MDS and t-SNE algorithms showed no separability between unharvested and harvested data for wheat, poor separability for maize and fair separability for soybean fields. In this example, the t-SNE algorithm showed more discriminatory power compared to MDS and PCA, indicating non-linearity in soybean data. The results of classification models based on the SVM and MLP algorithms were in line with the observed separability of data points in 2D space obtained by clustering. The best results of harvest detection were obtained for soybean, followed by maize and then wheat. Considering all performance metrics, both algorithms showed quite similar behaviour, having SVM slightly outperforming MLP. In the case of the regression models, the neural network-based algorithm, i.e., LSTM, showed better performance in the task of harvest date prediction compared to the RF algorithm, providing results with an MAE around six days, which is in line with the time interval of the acquisition of Sentinel-1 images. This result reflects the usability of the LSTM model in the studied region for operational purpose.

## Figures and Tables

**Table 1:** Hyper-parameters used in clustering.

Method	Parameters	All Crops
PCA	no. of components	2
MDS	no. of components	2
	max. no. of iterations	3000
	eps	1 × 10^−12^
	no. of initializations	1
t-SNE	no. of components	2
	random state	60
	perplexity	5

**Table 2:** Hyper-parameters used in classification models.

Method	Parameters	All Crops	Wheat	Maize	Soybean
SVM	kernel	linear	rbf	rbf	rbf
	C	1	10	1	1
	gamma	1	0.1	scale	scale
MLP	hidden layer size	(256,)	(128, 128)	(256,)	(128,)
	activation	identity	tanh	tanh	relu

**Table 3:** Hyper-parameters used in regression models.

Method	Parameters	All Crops
RF	no. of estimators	200
	max. depth	10
	min. samples to split	5
	min. samples at leaf	4
LSTM	hidden layer size	(128, 128)
	dropout	0
	activation	tanh

**Table 4:** Performance metrics of classification models.

Method	Metrics	All Crops	Wheat	Maize	Soybean
SVM	Acc	80.40	79.65	83.41	95.97
	P	82.74	72.08	90.24	95.63
	R	79.05	84.95	79.40	96.29
	F1	80.85	77.99	84.47	95.96
MLP	Acc	80.42	76.69	83.63	95.72
	P	81.23	75.82	88.67	95.89
	R	79.93	77.16	80.55	95.56
	F1	80.57	76.48	84.42	95.72

**Table 5:** Performance metrics of the general classification model evaluated on specific crops.

Method	Metrics	Wheat	Maize	Soybean
SVM	Acc	72.25	78.95	90.46
	P	72.85	79.48	90.81
	R	72.25	78.95	90.46
	F1	72.13	78.85	90.44
MLP	Acc	71.95	78.58	91.29
	P	72.77	79.06	91.54
	R	71.95	78.58	91.29
	F1	71.77	78.50	91.27

**Table 6:** Performance metrics of the best regression models.

Method	Metrics	All Crops	Wheat	Maize	Soybean
RF	$R^{2}$	0.62	0.19	0.46	−1.64
	RMSE	10.72	6.87	11.68	13.04
	MAE	8.55	5.95	9.78	10.11
LSTM	$R^{2}$	0.72	0.30	0.48	−0.04
	RMSE	9.25	5.86	11.41	9.56
	MAE	6.80	4.87	8.31	7.28

## Data Availability

Sentinel-1 SAR images used in the study are available on the Google Earth Engine platform. Geolocation of the fields and harvest dates used in the study are the property of Delta Agrar company and cannot be shared with the third parties due to an NDA.

## Supplementary Materials

The following supporting information can be downloaded at: https://www.mdpi.com/article/10.3390/s25072239/s1.

## Appendix A

**Table A1:** Performance metrics of classification models after the random permutation test.

Method	Metrics	A VH	A VV	D VH	D VV
SVM	Acc	56.49	76.15	70.31	79.83
	P	56.51	76.17	70.35	79.92
	R	56.49	76.15	70.31	79.83
	F1	56.49	76.15	70.30	79.82
MLP	Acc	57.39	76.40	70.29	79.64
	P	57.41	76.43	70.32	79.67
	R	57.39	76.40	70.29	79.64
	F1	57.37	76.40	70.29	79.63
