# Supplementary material for: Machine Learning-Based Harvest Date Detection and Prediction Using SAR Data for the Vojvodina Region (Serbia)
Source: Sensors (Basel). 2025 Apr 2;25(7):2239. doi: 10.3390/s25072239 (PMC11990955; doi:10.3390/s25072239)
Supplement: Supplementary file 1 [file sensors-25-02239-s001.zip › sensors-3466262-supplementary.pdf]

2017	
**Ascending**	**Descending**
03 May 2017<br>04 May 2017<br>05 May 2017	May 02 2017<br>May 04 2017
09 May 2017<br>10 May 2017<br>11 May 2017	May 08 2017<br>May 10 2017
15 May 2017<br>16 May 2017<br>17 May 2017	May 14 2017<br>May 15 2017<br>May 16 2017
21 May 2017<br>22 May 2017<br>23 May 2017	May 20 2017<br>May 21 2017<br>May 22 2017
27 May 2017<br>28 May 2017<br>29 May 2017	May 26 2017<br>May 27 2017<br>May 28 2017
02 June 2017<br>03 June 2017<br>04 June 2017	June 01 2017<br>June 02 2017<br>June 03 2017
08 June 2017<br>09 June 2017<br>10 June 2017	June 07 2017<br>June 08 2017<br>June 09 2017
14 June 2017<br>15 June 2017<br>16 June 2017	June 13 2017<br>June 14 2017<br>June 15 2017
20 June 2017<br>21 June 2017<br>22 June 2017	June 19 2017<br>June 20 2017<br>June 21 2017
26 June 2017<br>27 June 2017<br>28 June 2017	June 25 2017<br>June 26 2017<br>June 27 2017
03 July 2017<br>04 July 2017	July 01 2017<br>July 02 2017<br>July 03 2017
08 July 2017<br>09 July 2017<br>10 July 2017	July 07 2017<br>July 08 2017<br>July 09 2017
14 July 2017<br>15 July 2017<br>16 July 2017	July 13 2017<br>July 14 2017<br>July 15 2017
20 July 2017<br>21 July 2017<br>22 July 2017	July 19 2017<br>July 21 2017
26 July 2017<br>27 July 2017<br>28 July 2017	July 25 2017<br>July 26 2017
	July 31 2017

2018	
**Ascending**	**Descending**
04 May 2018<br>05 May 2018<br>06 May 2018	May 03 2018<br>May 04 2018<br>May 05 2018
10 May 2018<br>11 May 2018<br>12 May 2018	May 09 2018<br>May 10 2018<br>May 11 2018
16 May 2018<br>17 May 2018<br>18 May 2018	May 15 2018<br>May 16 2018<br>May 17 2018
22 May 2018<br>23 May 2018<br>24 May 2018	May 21 2018<br>May 22 2018<br>May 23 2018
28 May 2018<br>29 May 2018<br>30 May 2018	May 27 2018<br>May 28 2018<br>May 29 2018
03 June 2018<br>04 June 2018<br>05 June 2018	June 03 2018<br>June 04 2018
09 June 2018<br>10 June 2018<br>11 June 2018	June 08 2018<br>June 09 2018<br>June 10 2018
15 June 2018<br>16 June 2018<br>17 June 2018	June 14 2018<br>June 15 2018<br>June 16 2018
21 June 2018<br>22 June 2018<br>23 June 2018	June 20 2018<br>June 21 2018<br>June 22 2018
27 June 2018<br>28 June 2018	June 26 2018<br>June 27 2018<br>June 28 2018
03 July 2018<br>04 July 2018<br>05 July 2018	July 02 2018<br>July 03 2018<br>July 04 2018
09 July 2018<br>10 July 2018<br>11 July 2018	July 08 2018<br>July 09 2018<br>July 10 2018
15 July 2018<br>16 July 2018<br>17 July 2018	July 14 2018<br>July 15 2018<br>July 16 2018
21 July 2018<br>22 July 2018<br>23 July 2018	July 20 2018<br>July 21 2018<br>July 22 2018
27 July 2018<br>28 July 2018<br>29 July 2018	July 26 2018<br>July 27 2018<br>July 28 2018
15 August 2018<br>16 August 2018	15 August 2018
20 August 2018<br>21 August 2018<br>22 August 2018	19 August 2018<br>20 August 2018<br>21 August 2018
26 August 2018<br>27 August 2018<br>28 August 2018	25 August 2018<br>26 August 2018<br>27 August 2018
01 September 2018	31 August 2018

02 September 2018<br>03 September 2018	01 September 2018<br>02 September 2018
07 September 2018<br>08 September 2018<br>09 September 2018	06 September 2018<br>07 September 2018<br>08 September 2018
13 September 2018<br>14 September 2018<br>15 September 2018	12 September 2018<br>13 September 2018<br>14 September 2018
19 September 2018<br>20 September 2018<br>21 September 2018	18 September 2018<br>19 September 2018<br>20 September 2018
25 September 2018<br>26 September 2018<br>27 September 2018	24 September 2018<br>25 September 2018<br>26 September 2018
01 October 2018<br>02 October 2018<br>03 October 2018	30 September 2018<br>01 October 2018<br>02 October 2018
07 October 2018<br>08 October 2018<br>09 October 2018	06 October 2018<br>07 October 2018<br>08 October 2018
13 October 2018<br>14 October 2018<br>15 October 2018	12 October 2018<br>13 October 2018<br>14 October 2018
19 October 2018<br>20 October 2018<br>21 October 2018	18 October 2018<br>19 October 2018<br>20 October 2018
25 October 2018<br>26 October 2018<br>27 October 2018	24 October 2018<br>25 October 2018<br>26 October 2018
31 October 2018<br>01 November 2018<br>02 November 2018	30 October 2018<br>31 October 2018<br>01 November 2018
06 November 2018<br>07 November 2018<br>08 November 2018	05 November 2018<br>06 November 2018
12 November 2018<br>13 November 2018<br>14 November 2018	11 November 2018<br>12 November 2018<br>13 November 2018
18 November 2018<br>19 November 2018<br>20 November 2018	17 November 2018<br>18 November 2018<br>19 November 2018
24 November 2018<br>25 November 2018<br>26 November 2018	23 November 2018<br>24 November 2018<br>25 November 2018
30 November 2018	29 November 2018<br>30 November 2018

2019	
**Ascending**	**Descending**
01 May 2019	
05 May 2019<br>06 May 2019<br>07 May 2019	04 May 2019<br>05 May 2019<br>06 May 2019
11 May 2019<br>12 May 2019<br>13 May 2019	10 May 2019<br>11 May 2019<br>12 May 2019
17 May 2019<br>18 May 2019<br>19 May 2019	16 May 2019<br>17 May 2019<br>18 May 2019
23 May 2019<br>24 May 2019<br>25 May 2019	22 May 2019<br>23 May 2019<br>24 May 2019
29 May 2019<br>30 May 2019<br>31 May 2019	28 May 2019<br>29 May 2019<br>30 May 2019
04 June 2019<br>05 June 2019<br>06 June 2019	03 June 2019<br>04 June 2019<br>05 June 2019
10 June 2019<br>11 June 2019<br>12 June 2019	09 June 2019<br>10 June 2019<br>11 June 2019
16 June 2019<br>17 June 2019<br>18 June 2019	15 June 2019<br>16 June 2019<br>17 June 2019
22 June 2019<br>23 June 2019<br>24 June 2019	21 June 2019<br>22 June 2019<br>23 June 2019
28 June 2019<br>29 June 2019<br>30 June 2019	27 June 2019<br>28 June 2019
04 July 2019<br>05 July 2019<br>06 July 2019	03 July 2019<br>04 July 2019<br>05 July 2019
10 July 2019<br>11 July 2019<br>12 July 2019	09 July 2019<br>10 July 2019<br>11 July 2019
16 July 2019<br>17 July 2019<br>18 July 2019	15 July 2019<br>16 July 2019<br>17 July 2019
22 July 2019<br>23 July 2019<br>24 July 2019	21 July 2019<br>22 July 2019<br>23 July 2019
28 July 2019<br>29 July 2019<br>30 July 2019	27 July 2019<br>28 July 2019<br>29 July 2019
15 August 2019<br>16 August 2019<br>17 August 2019	15 August 2019<br>16 August 2019
21 August 2019<br>22 August 2019<br>23 August 2019	20 August 2019<br>21 August 2019<br>22 August 2019
27 August 2019<br>28 August 2019	26 August 2019<br>27 August 2019

29 August 2019	28 August 2019
02 September 2019<br>03 September 2019<br>04 September 2019	01 September 2019<br>02 September 2019<br>03 September 2019
08 September 2019<br>09 September 2019<br>10 September 2019	07 September 2019<br>08 September 2019<br>09 September 2019
14 September 2019<br>15 September 2019<br>16 September 2019	13 September 2019<br>14 September 2019<br>15 September 2019
20 September 2019<br>21 September 2019<br>22 September 2019	19 September 2019<br>20 September 2019<br>21 September 2019
26 September 2019<br>27 September 2019<br>28 September 2019	25 September 2019<br>27 September 2019
02 October 2019<br>03 October 2019<br>04 October 2019	01 October 2019<br>02 October 2019<br>03 October 2019
08 October 2019<br>09 October 2019<br>10 October 2019	07 October 2019<br>08 October 2019<br>09 October 2019
14 October 2019<br>15 October 2019<br>16 October 2019	13 October 2019<br>14 October 2019<br>15 October 2019
20 October 2019<br>21 October 2019<br>22 October 2019	19 October 2019<br>20 October 2019<br>21 October 2019
26 October 2019<br>27 October 2019<br>28 October 2019	25 October 2019<br>26 October 2019<br>27 October 2019
01 November 2019<br>02 November 2019<br>03 November 2019	31 October 2019<br>01 November 2019<br>02 November 2019
07 November 2019<br>08 November 2019<br>09 November 2019	06 November 2019<br>07 November 2019<br>08 November 2019
13 November 2019<br>14 November 2019<br>15 November 2019	13 November 2019<br>14 November 2019
19 November 2019<br>20 November 2019<br>21 November 2019	18 November 2019<br>19 November 2019<br>20 November 2019
25 November 2019<br>26 November 2019<br>27 November 2019	24 November 2019<br>25 November 2019<br>26 November 2019
	30 November 2019

2020	
**Ascending**	**Descending**
May 01 2020	
May 06 2020<br>May 07 2020	May 4 2020<br>May 5 2020<br>May 6 2020
May 12 2020<br>May 13 2020	May 10 2020<br>May 11 2020<br>May 12 2020
May 18 2020<br>May 19 2020	May 16 2020<br>May 17 2020<br>May 18 2020
May 24 2020<br>May 25 2020	May 22 2020<br>May 23 2020<br>May 24 2020
May 30 2020<br>May 31 2020	May 28 2020<br>May 29 2020<br>May 30 2020
June 05 2020<br>June 06 2020	June 3 2020<br>June 4 2020<br>June 5 2020
June 11 2020<br>June 12 2020	June 9 2020<br>June 10 2020<br>June 11 2020
June 17 2020<br>June 18 2020	June 15 2020<br>June 16 2020<br>June 17 2020
June 23 2020<br>June 24 2020	June 21 2020<br>June 22 2020<br>June 23 2020
June 29 2020<br>June 30 2020	June 27 2020<br>June 28 2020<br>June 29 2020
July 05 2020<br>July 06 2020	July 3 2020<br>July 4 2020<br>July 5 2020
July 11 2020<br>July 12 2020	July 9 2020<br>July 10 2020<br>July 11 2020
July 17 2020<br>July 18 2020	July 15 2020<br>July 16 2020<br>July 17 2020
July 23 2020<br>July 24 2020	July 21 2020<br>July 22 2020<br>July 23 2020
July 29 2020<br>July 30 2020	July 27 2020<br>July 28 2020<br>July 29 2020
August 15 2020<br>August 16 2020<br>August 17 2020	August 15 2020<br>August 16 2020
August 21 2020<br>August 22 2020<br>August 23 2020	August 20 2020<br>August 21 2020<br>August 22 2020
August 27 2020<br>August 28 2020	August 26 2020<br>August 27 2020

August 29 2020	August 28 2020
September 02 2020<br>September 03 2020<br>September 04 2020	September 1 2020<br>September 2 2020<br>September 3 2020
September 08 2020<br>September 09 2020<br>September 10 2020	September 7 2020<br>September 8 2020<br>September 9 2020
September 14 2020<br>September 15 2020<br>September 16 2020	September 13 2020<br>September 14 2020<br>September 15 2020
September 20 2020<br>September 21 2020<br>September 22 2020	September 19 2020<br>September 20 2020<br>September 21 2020
September 26 2020<br>September 27 2020<br>September 28 2020	September 25 2020<br>September 26 2020<br>September 27 2020
October 02 2020<br>October 03 2020<br>October 04 2020	October 1 2020<br>October 2 2020<br>October 3 2020
October 08 2020<br>October 09 2020<br>October 10 2020	October 8 2020<br>October 9 2020
October 14 2020<br>October 15 2020<br>October 16 2020	October 13 2020<br>October 14 2020<br>October 15 2020
October 20 2020<br>October 21 2020<br>October 22 2020	October 19 2020<br>October 20 2020<br>October 21 2020
October 26 2020<br>October 27 2020<br>October 28 2020	October 25 2020<br>October 26 2020<br>October 27 2020
November 01 2020<br>November 02 2020<br>November 03 2020	October 31 2020<br>November 1 2020<br>November 2 2020
November 07 2020<br>November 08 2020<br>November 09 2020	November 6 2020<br>November 7 2020<br>November 8 2020
November 13 2020<br>November 14 2020<br>November 15 2020	November 12 2020<br>November 13 2020<br>November 14 2020
November 19 2020<br>November 20 2020<br>November 21 2020	November 18 2020<br>November 19 2020<br>November 20 2020
November 25 2020<br>November 26 2020<br>November 27 2020	November 24 2020<br>November 25 2020<br>November 26 2020
	November 30 2020
